# Fall prevention interventions for Hispanic/Latino older adults: a scoping review protocol

**DOI:** 10.3389/fpubh.2025.1615837

**Published:** 2025-07-02

**Authors:** Janet Lopez, Michael Joseph Dino, Chitra Banarjee, Ladda Thiamwong

**Affiliations:** ^1^College of Nursing, University of Central Florida, Orlando, FL, United States; ^2^College of Medicine, University of Central Florida, Orlando, FL, United States

**Keywords:** Hispanic/Latino, older adults, fall prevention, interventions, United States

## Abstract

**Background:**

Despite the importance of fall prevention among older adults, limited research exists on interventions tailored to rapidly growing Hispanic/Latino populations in the United States. This protocol paper outlines the rationale and methods for conducting a scoping review to address this knowledge gap.

**Methods and analysis:**

We will follow Arksey and O’Malley’s framework and Preferred Reporting Items for Systematic Reviews and Meta-Analysis extension for scoping reviews (PRISMA-ScR). This scoping review will be guided by the following research questions: What characterizes fall prevention interventions for Hispanic/Latino older adults? An iterative and systematic search of peer-reviewed publications from four databases (PubMed, PsycINFO, Web of Science, and CINAHL) will be extracted and uploaded to Covidence for screening.

**Trial registration:**

This scoping review protocol has been registered with Open Science Framework and can be accessed at https://osf.io/zx3m8.

## Highlights

- This protocol outlines a comprehensive approach for conducting a scoping review of fall prevention interventions intended for an unrepresented cohort of older adults.
- Using well-established guidelines, theories, and frameworks is paramount to the success of this review.
- The study design allows feasibility assessment and outlines potential issues for implementation and sustainability.
- The scoping review findings may reveal gaps in the existing literature, guiding targeted studies or clinical trials for fall prevention in Hispanic/Latino older adults.
- The scoping review may miss relevant studies due to database selection.
- Focusing on studies published in the United States will present a limitation, however this was necessary given the variation in health delivery models across countries.
- The scoping review is limited by the inclusion of studies that did not specifically target Hispanic older adults, which may obscure the unique challenges and effective interventions for this population.
- The study will aim for a comprehensive literature review using a scoping strategy rather than adhering to specific standards of evidence. This approach may be considered a limitation.

## 1 Introduction

Falls are a significant public health concern for older adults (≥ 60 years) due to the risk of serious injury and death (1). The aging Hispanic/Latino population is rapidly growing in the U. S. (2, 3). There is a lack of fall prevention research tailored for Spanish-speaking Hispanic/Latino older adults (4). Culturally and linguistically appropriate fall prevention programs are essential for addressing the gap in perceived fall risk and fear of falling among Hispanic/Latino older adults. Physical activity may be an effective and accessible mechanism to reduce age-related risks associated with falls and fall injuries (5). However, Hispanic/Latino older adults are less likely to participate in leisure time physical activity compared to non-Hispanic/Latino white older adults due to multifactorial barriers including low socioeconomic status, lack of bilingual or bicultural programs, limited resources, perceived safety, and time constraints (4–7). Understanding fall prevention among Hispanic/Latino older adults is crucial for developing culturally tailored interventions. Falls are a significant health concern for this population, which often has limited access to resources. By improving our understanding of their unique needs, we can enhance access to effective fall prevention strategies.

## 2 Methods and analysis

**Figure 1:**
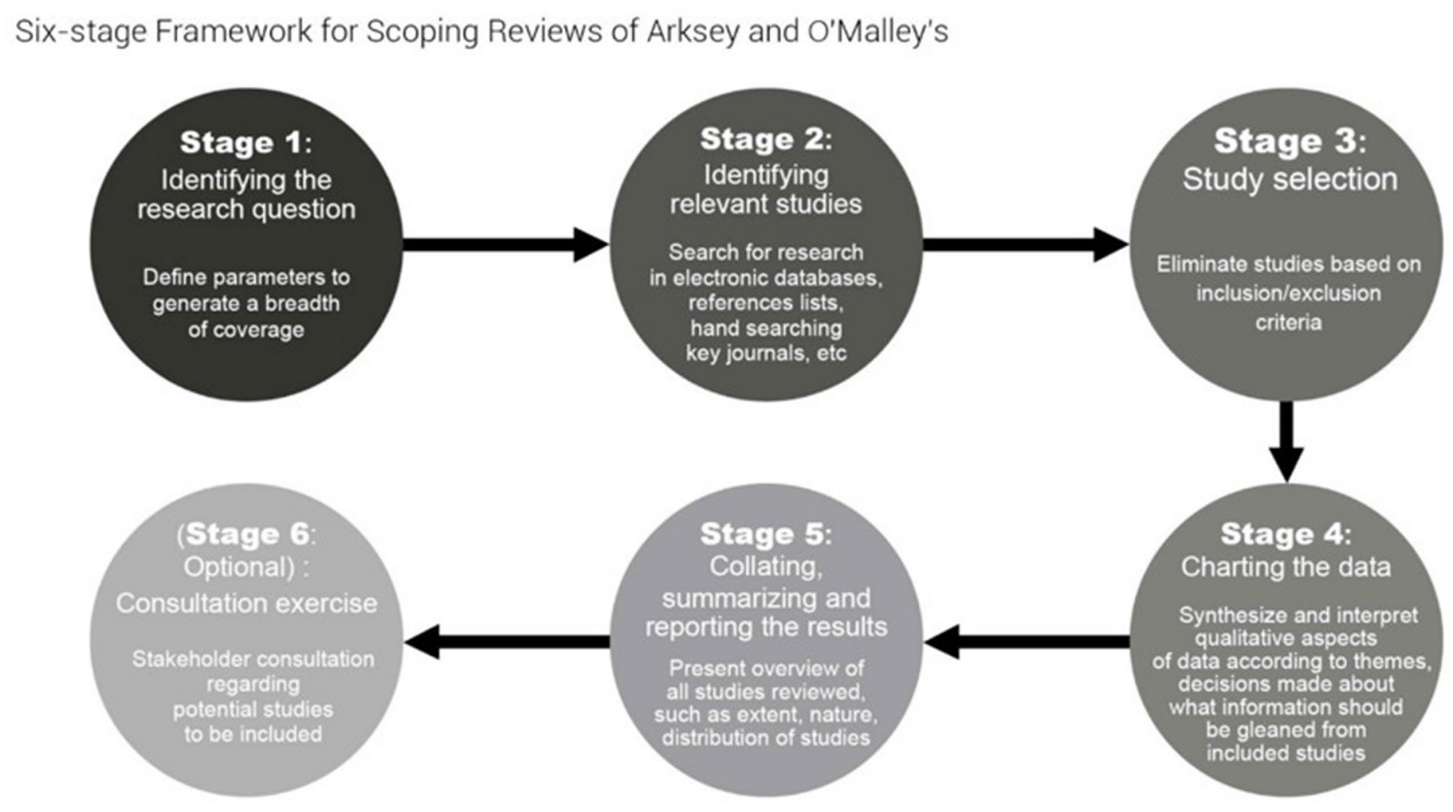
Schematic of Arksey and O′Malley’s (9, 10) six stage framework for scoping reviews.

This scoping review will follow the stages outlined by Arksey and O’Malley framework (8), along with updates made to improve their method (9, 10). This process involves six stages: formulating research questions, identifying relevant studies, selecting studies based on criteria, charting data, summarizing results, and consulting stakeholders. (See Figure 1 Schematic Arksey and O’Malley framework’s six steps framework for scoping review) We will adhere to the Preferred Reporting Items for Systematic Reviews and Meta-Analysis extension for scoping reviews (PRISMA-ScR) (11). The process will involve refining the search, reviewing articles, and collecting relevant data. The protocol for the scoping review has been registered with the Open Science Framework (osf.io/zx3m8). Any changes to the protocol will be specified in the methods section of the published review.

### 2.1 Stage 1: formulating the research question

Research questions were formulated by considering four key elements: fall risk management (concept), Hispanic/Latino older adults aged 60 years and older (target population), community settings (context), and the range of outcomes measured in fall intervention programs. This approach helped to refine the focus of the review and create a more effective search strategy. Consistent with our objectives, this scoping review will be guided by the following research questions and sub-questions:

1. What characterizes fall prevention intervention for Hispanic/Latino older adults?
  1.1 What intervention models are utilized for fall prevention among Hispanic/Latino older adults?
  1.2 What methods and outcomes are associated with fall prevention interventions in Hispanic/Latino older adults?
  1.3 What are the facilitators and barriers to implementing fall prevention interventions among Hispanic older adults?
  1.4 How are cultural adaptations (e.g., language, location, traditions) integrated into the design and implementation of fall prevention interventions for Hispanic/Latino older adults?

### 2.2 Stage 2: identifying relevant studies

To help identify pertinent search terms and databases, we consulted with an experienced subject librarian from the University of Central Florida, College of Medicine. A group of relevant stakeholders, including a fall prevention expert, Hispanic/Latino older adults, and community leaders, have reviewed the search terms. An initial search will be executed across select databases to capture relevant studies. This will involve an examination of the keywords present in the titles and abstracts, as well as the index terms assigned to each article. A more extensive search will be performed utilizing the identified keywords and index terms, encompassing four key databases: PubMed, PsycINFO, CINAHL, and Web of Science. We will conduct backward searches, meaning that we will examine the reference lists of all included articles to find additional studies. The search will be limited to articles published within English studies in the United States. We decided not to limit the date range for this scoping review to conduct a thorough investigation of the topic, which may have limited published studies. As such, we will include all relevant studies, regardless of the publication date, to get a complete picture of the literature.

The initial search strategy was developed using PubMed and will be adapted for use with additional databases (refer to Table 1). We would like to note that we utilized the Medical Library Association Latinx Caucus’s Latinx/Hispanic US population search hedges to guide our search for the target population (12). The search will utilize a combination of subject headings and keywords, employing Boolean logic and operators (e.g., ‘AND,’ ‘OR’) to refine the results.

**Table 1:** Database search strategy.

Database (number of articles)	Search strategy
Medline-PubMed (139)	(((((((((((“fall* prevention”) OR (“fall* intervention*”)) OR (“injury prevention*”)) OR (“fall management”)) OR (“peer-led fall prevention”)) OR (“evidence-based fall* prevention*”)) OR (“fall risk reduction”)) OR (“accidental fall* prevention*”)) OR (“Accidental Falls”[Mesh])) AND (((((((workshop*[tiab]) OR (campaign*[tiab])) OR (service*)) OR (training*[tiab])) OR (strateg*)) OR (awareness[tiab])) OR (“education” [Subheading]))) AND (((“Hispanic or Latino”[Mesh] OR Hispanic*[tiab] OR hispano*[tiab] OR hispana*[tiab] OR Latino*[tiab] OR Latina*[tiab] OR Latine*[tiab] OR Latinu*[tiab] OR Latinx[tiab] OR “Spanish American*”[tiab] OR “Mexican American*”[tiab] OR Chicana*[tiab] OR Chicano*[tiab] OR Chicanx[tiab] OR “Puerto Rico”[Mesh] OR “Puerto Rico”[tiab] OR “Puerto Rican*”[tiab] OR Boricua*[tiab] OR “Cuban American*”[tiab]) OR ((“Spanish speak*”[tiab] OR Argentina[Mesh] OR Argentina[tiab] OR Argentine*[tiab] OR Argentinian*[tiab] OR Bolivia[Mesh] OR Bolivia[tiab] OR Bolivian*[tiab] OR Brazil[Mesh] OR Brazil[tiab] OR Brazilian*[tiab] OR Chile[Mesh] OR Chile[tiab] OR Chilean*[tiab] OR Colombia[Mesh] OR Colombia[tiab] OR Colombian*[tiab] OR “Costa Rica”[Mesh] OR “Costa Rica”[tiab] OR “Costa Rican*”[tiab] OR Cuba[Mesh] OR Cuba[tiab] OR Cuban*[tiab] OR “Hispaniola”[tiab] OR “Dominican Republic”[Mesh] OR Dominican*[tiab] OR Ecuador[Mesh] OR Ecuador[tiab] OR Ecuadorian*[tiab] OR Ecuadorean*[tiab] OR “El Salvador”[Mesh] OR “El Salvador”[tiab] OR Salvadorean*[tiab] OR Salvadoran*[tiab] OR Salvadorian*[tiab] OR Guatemala[Mesh] OR Guatemala[tiab] OR Guatemalan*[tiab] OR Honduras[Mesh] OR Honduras[tiab] OR Honduran*[tiab] OR Mexico[Mesh] OR Mexico[tiab] OR Mexican*[tiab] OR Nicaragua[Mesh] OR Nicaragua[tiab] OR Nicaraguan*[tiab] OR Panama[Mesh] OR Panama[tiab] OR Panamanian*[tiab] OR Paraguay[Mesh] OR Paraguay[tiab] OR Paraguayan*[tiab] OR “Peru”[Mesh] OR Peru[tiab] OR Peruvian*[tiab] OR “Uruguay”[Mesh] OR Uruguay[tiab] OR Uruguayan*[tiab] OR “Venezuela”[Mesh] OR Venezuela[tiab] OR Venezuelan*[tiab] OR “Central America”[Mesh: NoExp] OR “Central America*”[tiab] OR “Caribbean Region”[Mesh: NoExp] OR Caribbean*[tiab] OR “South America”[Mesh: NoExp] OR “South America*”[tiab] OR “Latin America”[Mesh] OR “Latin America*”[tiab]) AND (“United States”[Mesh] OR “United State*”[tiab] OR “USA”[tiab] OR “North America”[Mesh: NoExp] OR Appalachia*[tiab] OR “great lakes”[tiab] OR “mid atlantic state*”[tiab] OR “mid atlantic region*”[tiab] OR “middle atlantic state*”[tiab] OR “middle atlantic region*”[tiab] OR “midwestern us”[tiab] OR “midwestern state*”[tiab] OR “midwest state*”[tiab] OR “midwest us”[tiab] OR “great plains”[tiab] OR “heartland”[tiab] OR “new england”[tiab] OR “northeastern us”[tiab] OR “northeastern state*”[tiab] OR “northeast state*”[tiab] OR “northeast us”[tiab] OR “pacific northwest”[tiab] OR (northwest*[tiab] AND “us”[tiab]) OR “northwestern state*”[tiab] OR “northwest state*”[tiab] OR “pacific state*”[tiab] OR “southeast state*”[tiab] OR “southeastern state*”[tiab] OR “southeast us”[tiab] OR “southeastern us”[tiab] OR “southern state*”[tiab] OR “southern us”[tiab] OR “southwest state*”[tiab] OR “southwestern state*”[tiab] OR “southwest us”[tiab] OR “southwestern us”[tiab] OR “deep south”[tiab] OR “black belt”[tiab] OR “rust belt”[tiab] OR “District of Columbia”[tiab] OR “Washington DC”[tiab] OR Alabama[tiab] OR Alaska[tiab] OR Arizona[tiab] OR Arkansas[tiab] OR California[tiab] OR Colorado[tiab] OR Connecticut[tiab] OR Delaware[tiab] OR Florida[tiab] OR Georgia[tiab] OR Hawaii[tiab] OR “Hawai i”[tiab] OR Idaho[tiab] OR Illinois[tiab] OR Indiana[tiab] OR Iowa[tiab] OR Kansas[tiab] OR Kentucky[tiab] OR Louisiana[tiab] OR Maine[tiab] OR Maryland[tiab] OR Massachusetts[tiab] OR Michigan[tiab] OR Minnesota[tiab] OR Mississippi[tiab] OR Missouri[tiab] OR Montana[tiab] OR Nebraska[tiab] OR Nevada[tiab] OR “New Hampshire”[tiab] OR “New Jersey”[tiab] OR “New Mexico”[tiab] OR “New York”[tiab] OR “North Carolina”[tiab] OR “North Dakota”[tiab] OR Ohio[tiab] OR Oklahoma[tiab] OR Oregon[tiab] OR Pennsylvania[tiab] OR “Rhode Island”[tiab] OR “South Carolina”[tiab] OR “South Dakota”[tiab] OR Tennessee[tiab] OR Texas[tiab] OR Utah[tiab] OR Vermont[tiab] OR Virginia[tiab] OR Washington[tiab] OR “West Virginia”[tiab] OR Wisconsin[tiab] OR Wyoming[tiab] OR “Western Hemisphere”[tiab] OR “Americas”[Mesh: NoExp] OR America*[tiab]))))) AND ((((((((((((“older adult*”[tiab]) OR (frail[tiab])) OR (aged[tiab])) OR (elderly[tiab])) OR (elder[tiab])) OR (senior*[tiab])) OR (gerontology[tiab])) OR (geriatrics[tiab])) OR (geria*[tiab])) OR (“Aged”[Mesh])) OR (“Frail Elderly”[Mesh])) OR (“Geriatrics”[Mesh]))
CINAHL (108)	(((((((((((“fall* prevention”) OR (“fall* intervention*”)) OR (“injury prevention*”)) OR (“fall management”)) OR (“peer-led fall prevention”)) OR (“evidence-based fall* prevention*”)) OR (“fall risk reduction”)) OR (“accidental fall* prevention*”)) OR ((MH “Accidental Falls+”))) AND ((((((((TI workshop* OR AB workshop*)) OR ((TI campaign* OR AB campaign*))) OR (service*)) OR ((TI training* OR AB training*))) OR (strateg*)) OR ((TI awareness OR AB awareness))) OR (“education [Subheading]”))) AND ((((MH “Hispanic or Latino+”) OR (TI Hispanic* OR AB Hispanic*) OR (TI hispano* OR AB hispano*) OR (TI hispana* OR AB hispana*) OR (TI Latino* OR AB Latino*) OR (TI Latina* OR AB Latina*) OR (TI Latine* OR AB Latine*) OR (TI Latinu* OR AB Latinu*) OR (TI Latinx OR AB Latinx) OR (TI “Spanish American*” OR AB “Spanish American*”) OR (TI “Mexican American*” OR AB “Mexican American*”) OR (TI Chicana* OR AB Chicana*) OR (TI Chicano* OR AB Chicano*) OR (TI Chicanx OR AB Chicanx) OR (MH “Puerto Rico+”) OR (TI “Puerto Rico” OR AB “Puerto Rico”) OR (TI “Puerto Rican*” OR AB “Puerto Rican*”) OR (TI Boricua* OR AB Boricua*) OR (TI “Cuban American*” OR AB “Cuban American*”)) OR (((TI “Spanish speak*” OR AB “Spanish speak*”) OR (MH Argentina+) OR (TI Argentina OR AB Argentina) OR (TI Argentine* OR AB Argentine*) OR (TI Argentinian* OR AB Argentinian*) OR (MH Bolivia+) OR (TI Bolivia OR AB Bolivia) OR (TI Bolivian* OR AB Bolivian*) OR (MH Brazil+) OR (TI Brazil OR AB Brazil) OR (TI Brazilian* OR AB Brazilian*) OR (MH Chile+) OR (TI Chile OR AB Chile) OR (TI Chilean* OR AB Chilean*) OR (MH Colombia+) OR (TI Colombia OR AB Colombia) OR (TI Colombian* OR AB Colombian*) OR (MH “Costa Rica+”) OR (TI “Costa Rica” OR AB “Costa Rica”) OR (TI “Costa Rican*” OR AB “Costa Rican*”) OR (MH Cuba+) OR (TI Cuba OR AB Cuba) OR (TI Cuban* OR AB Cuban*) OR (TI Hispaniola OR AB Hispaniola) OR (MH “Dominican Republic+”) OR (TI Dominican* OR AB Dominican*) OR (MH Ecuador+) OR (TI Ecuador OR AB Ecuador) OR (TI Ecuadorian* OR AB Ecuadorian*) OR (TI Ecuadorean* OR AB Ecuadorean*) OR (MH “El Salvador+”) OR (TI “El Salvador” OR AB “El Salvador”) OR (TI Salvadorean* OR AB Salvadorean*) OR (TI Salvadoran* OR AB Salvadoran*) OR (TI Salvadorian* OR AB Salvadorian*) OR (MH Guatemala+) OR (TI Guatemala OR AB Guatemala) OR (TI Guatemalan* OR AB Guatemalan*) OR (MH Honduras+) OR (TI Honduras OR AB Honduras) OR (TI Honduran* OR AB Honduran*) OR (MH Mexico+) OR (TI Mexico OR AB Mexico) OR (TI Mexican* OR AB Mexican*) OR (MH Nicaragua+) OR (TI Nicaragua OR AB Nicaragua) OR (TI Nicaraguan* OR AB Nicaraguan*) OR (MH Panama+) OR (TI Panama OR AB Panama) OR (TI Panamanian* OR AB Panamanian*) OR (MH Paraguay+) OR (TI Paraguay OR AB Paraguay) OR (TI Paraguayan* OR AB Paraguayan*) OR (MH Peru+) OR (TI Peru OR AB Peru) OR (TI Peruvian* OR AB Peruvian*) OR (MH Uruguay+) OR (TI Uruguay OR AB Uruguay) OR (TI Uruguayan* OR AB Uruguayan*) OR (MH Venezuela+) OR (TI Venezuela OR AB Venezuela) OR (TI Venezuelan* OR AB Venezuelan*) OR (MH “Central America”) OR (TI “Central America*” OR AB “Central America*”) OR (MH “Caribbean Region”) OR (TI Caribbean* OR AB Caribbean*) OR (MH “South America”) OR (TI “South America*” OR AB “South America*”) OR (MH “Latin America+”) OR (TI “Latin America*” OR AB “Latin America*”)) AND ((MH “United States+”) OR (TI “United State*” OR AB “United State*”) OR (TI USA OR AB USA) OR (MH “North America”) OR (TI Appalachia* OR AB Appalachia*) OR (TI “great lakes” OR AB “great lakes”) OR (TI “mid atlantic state*” OR AB “mid atlantic state*”) OR (TI “mid atlantic region*” OR AB “mid atlantic region*”) OR (TI “middle atlantic state*” OR AB “middle atlantic state*”) OR (TI “middle atlantic region*” OR AB “middle atlantic region*”) OR (TI “midwestern us” OR AB “midwestern us”) OR (TI “midwestern state*” OR AB “midwestern state*”) OR (TI “midwest state*” OR AB “midwest state*”) OR (TI “midwest us” OR AB “midwest us”) OR (TI “great plains” OR AB “great plains”) OR (TI heartland OR AB heartland) OR (TI “new england” OR AB “new england”) OR (TI “northeastern us” OR AB “northeastern us”) OR (TI “northeastern state*” OR AB “northeastern state*”) OR (TI “northeast state*” OR AB “northeast state*”) OR (TI “northeast us” OR AB “northeast us”) OR (TI “pacific northwest” OR AB “pacific northwest”) OR ((TI northwest* OR AB northwest*) AND (TI us OR AB us)) OR (TI “northwestern state*” OR AB “northwestern state*”) OR (TI “northwest state*” OR AB “northwest state*”) OR (TI “pacific state*” OR AB “pacific state*”) OR (TI “southeast state*” OR AB “southeast state*”) OR (TI “southeastern state*” OR AB “southeastern state*”) OR (TI “southeast us” OR AB “southeast us”) OR (TI “southeastern us” OR AB “southeastern us”) OR (TI “southern state*” OR AB “southern state*”) OR (TI “southern us” OR AB “southern us”) OR (TI “southwest state*” OR AB “southwest state*”) OR (TI “southwestern state*” OR AB “southwestern state*”) OR (TI “southwest us” OR AB “southwest us”) OR (TI “southwestern us” OR AB “southwestern us”) OR (TI “deep south” OR AB “deep south”) OR (TI “black belt” OR AB “black belt”) OR (TI “rust belt” OR AB “rust belt”) OR (TI “District of Columbia” OR AB “District of Columbia”) OR (TI “Washington DC” OR AB “Washington DC”) OR (TI Alabama OR AB Alabama) OR (TI Alaska OR AB Alaska) OR (TI Arizona OR AB Arizona) OR (TI Arkansas OR AB Arkansas) OR (TI California OR AB California) OR (TI Colorado OR AB Colorado) OR (TI Connecticut OR AB Connecticut) OR (TI Delaware OR AB Delaware) OR (TI Florida OR AB Florida) OR (TI Georgia OR AB Georgia) OR (TI Hawaii OR AB Hawaii) OR (TI “Hawai i” OR AB “Hawai i”) OR (TI Idaho OR AB Idaho) OR (TI Illinois OR AB Illinois) OR (TI Indiana OR AB Indiana) OR (TI Iowa OR AB Iowa) OR (TI Kansas OR AB Kansas) OR (TI Kentucky OR AB Kentucky) OR (TI Louisiana OR AB Louisiana) OR (TI Maine OR AB Maine) OR (TI Maryland OR AB Maryland) OR (TI Massachusetts OR AB Massachusetts) OR (TI Michigan OR AB Michigan) OR (TI Minnesota OR AB Minnesota) OR (TI Mississippi OR AB Mississippi) OR (TI Missouri OR AB Missouri) OR (TI Montana OR AB Montana) OR (TI Nebraska OR AB Nebraska) OR (TI Nevada OR AB Nevada) OR (TI “New Hampshire” OR AB “New Hampshire”) OR (TI “New Jersey” OR AB “New Jersey”) OR (TI “New Mexico” OR AB “New Mexico”) OR (TI “New York” OR AB “New York”) OR (TI “North Carolina” OR AB “North Carolina”) OR (TI “North Dakota” OR AB “North Dakota”) OR (TI Ohio OR AB Ohio) OR (TI Oklahoma OR AB Oklahoma) OR (TI Oregon OR AB Oregon) OR (TI Pennsylvania OR AB Pennsylvania) OR (TI “Rhode Island” OR AB “Rhode Island”) OR (TI “South Carolina” OR AB “South Carolina”) OR (TI “South Dakota” OR AB “South Dakota”) OR (TI Tennessee OR AB Tennessee) OR (TI Texas OR AB Texas) OR (TI Utah OR AB Utah) OR (TI Vermont OR AB Vermont) OR (TI Virginia OR AB Virginia) OR (TI Washington OR AB Washington) OR (TI “West Virginia” OR AB “West Virginia”) OR (TI Wisconsin OR AB Wisconsin) OR (TI Wyoming OR AB Wyoming) OR (TI “Western Hemisphere” OR AB “Western Hemisphere”) OR (MH Americas) OR (TI America* OR AB America*)))))) AND (((((((((((((TI “older adult*” OR AB “older adult*”)) OR ((TI frail OR AB frail))) OR ((TI aged OR AB aged))) OR ((TI elderly OR AB elderly))) OR ((TI elder OR AB elder))) OR ((TI senior* OR AB senior*))) OR ((TI gerontology OR AB gerontology))) OR ((TI geriatrics OR AB geriatrics))) OR ((TI geria* OR AB geria*))) OR ((MH Aged+))) OR ((MH “Frail Elderly+”))) OR ((MH Geriatrics+)))
Web of Science (132)	(((TS = (“fall* prevention” OR “fall* intervention*” OR “injury prevention*” OR “fall management” OR “peer-led fall prevention” OR “evidence-based fall* prevention*” OR “fall risk reduction” OR “accidental fall* prevention*” OR “Accidental Falls”)) AND TS = (workshop* OR campaign* OR service* OR training* OR strateg* OR awareness OR education)) AND (((TS = (“Hispanic or Latino” OR Hispanic* OR hispano* OR hispana* OR Latino* OR Latina* OR Latine* OR Latinu* OR Latinx OR “Spanish American*” OR “Mexican American*” OR Chicana* OR Chicano* OR Chicanx OR “Puerto Rico” OR “Puerto Rican*” OR Boricua* OR “Cuban American*”)) OR TS = (“Spanish speak*” OR Argentina OR Argentine* OR Argentinian* OR Bolivia OR Bolivian* OR Brazil OR Brazilian* OR Chile OR Chilean* OR Colombia OR Colombian* OR “Costa Rica” OR “Costa Rican*” OR Cuba OR Cuban* OR Hispaniola OR “Dominican Republic” OR Dominican* OR Ecuador OR Ecuadorian* OR Ecuadorean* OR “El Salvador” OR Salvadorean* OR Salvadoran* OR Salvadorian* OR Guatemala OR Guatemalan* OR Honduras OR Honduran* OR Mexico OR Mexican* OR Nicaragua OR Nicaraguan* OR Panama OR Panamanian* OR Paraguay OR Paraguayan* OR Peru OR Peruvian* OR Uruguay OR Uruguayan* OR Venezuela OR Venezuelan* OR “Central America” OR “Central America*” OR “Caribbean Region” OR Caribbean* OR “South America” OR “South America*” OR “Latin America” OR “Latin America*”)) AND TS = (“United States” OR “United State*” OR USA OR “North America” OR Appalachia* OR “great lakes” OR “mid atlantic state*” OR “mid atlantic region*” OR “middle atlantic state*” OR “middle atlantic region*” OR “midwestern us” OR “midwestern state*” OR “midwest state*” OR “midwest us” OR “great plains” OR heartland OR “new england” OR “northeastern us” OR “northeastern state*” OR “northeast state*” OR “northeast us” OR “pacific northwest” OR “northwest* us” OR “northwestern state*” OR “northwest state*” OR “pacific state*” OR “southeast state*” OR “southeastern state*” OR “southeast us” OR “southeastern us” OR “southern state*” OR “southern us” OR “southwest state*” OR “southwestern state*” OR “southwest us” OR “southwestern us” OR “deep south” OR “black belt” OR “rust belt” OR “District of Columbia” OR “Washington DC” OR Alabama OR Alaska OR Arizona OR Arkansas OR California OR Colorado OR Connecticut OR Delaware OR Florida OR Georgia OR Hawaii OR “Hawai i” OR Idaho OR Illinois OR Indiana OR Iowa OR Kansas OR Kentucky OR Louisiana OR Maine OR Maryland OR Massachusetts OR Michigan OR Minnesota OR Mississippi OR Missouri OR Montana OR Nebraska OR Nevada OR “New Hampshire” OR “New Jersey” OR “New Mexico” OR “New York” OR “North Carolina” OR “North Dakota” OR Ohio OR Oklahoma OR Oregon OR Pennsylvania OR “Rhode Island” OR “South Carolina” OR “South Dakota” OR Tennessee OR Texas OR Utah OR Vermont OR Virginia OR Washington OR “West Virginia” OR Wisconsin OR Wyoming OR “Western Hemisphere” OR Americas OR America*))) AND TS = (“older adult*” OR frail OR aged OR elderly OR elder OR senior* OR gerontology OR geriatrics OR geria* OR “frail elderly”)
APA PsycInfo (66)	(TI “fall* prevention” OR TI “fall* intervention*” OR TI “injury prevention*” OR TI “fall management” OR TI “peer-led fall prevention” OR TI “evidence-based fall* prevention*” OR TI “fall risk reduction” OR TI “accidental fall* prevention*” OR MA “Accidental Falls” OR AB “fall* prevention” OR AB “fall* intervention*” OR AB “injury prevention*” OR AB “fall management” OR AB “peer-led fall prevention” OR AB “evidence-based fall* prevention*” OR AB “fall risk reduction” OR AB “accidental fall* prevention*”) AND (TI workshop* OR TI campaign* OR service* OR TI training* OR strateg* OR TI awareness OR MM education OR AB workshop* OR AB campaign* OR AB training* OR AB awareness) AND ((MA “Hispanic or Latino” OR TI Hispanic* OR TI hispano* OR TI hispana* OR TI Latino* OR TI Latina* OR TI Latine* OR TI Latinu* OR TI Latinx OR TI “Spanish American*” OR TI “Mexican American*” OR TI Chicana* OR TI Chicano* OR TI Chicanx OR MA “Puerto Rico” OR TI “Puerto Rico” OR TI “Puerto Rican*” OR TI Boricua* OR TI “Cuban American*” OR AB Hispanic* OR AB hispano* OR AB hispana* OR AB Latino* OR AB Latina* OR AB Latine* OR AB Latinu* OR AB Latinx OR AB “Spanish American*” OR AB “Mexican American*” OR AB Chicana* OR AB Chicano* OR AB Chicanx OR AB “Puerto Rico” OR AB “Puerto Rican*” OR AB Boricua* OR AB “Cuban American*”) OR (TI “Spanish speak*” OR MA Argentina OR TI Argentina OR TI Argentine* OR TI Argentinian* OR MA Bolivia OR TI Bolivia OR TI Bolivian* OR MA Brazil OR TI Brazil OR TI Brazilian* OR MA Chile OR TI Chile OR TI Chilean* OR MA Colombia OR TI Colombia OR TI Colombian* OR MA “Costa Rica” OR TI “Costa Rica” OR TI “Costa Rican*” OR MA Cuba OR TI Cuba OR TI Cuban* OR TI “Hispaniola” OR MA “Dominican Republic” OR TI Dominican* OR MA Ecuador OR TI Ecuador OR TI Ecuadorian* OR TI Ecuadorean* OR MA “El Salvador” OR TI “El Salvador” OR TI Salvadorean* OR TI Salvadoran* OR TI Salvadorian* OR MA Guatemala OR TI Guatemala OR TI Guatemalan* OR MA Honduras OR TI Honduras OR TI Honduran* OR MA Mexico OR TI Mexico OR TI Mexican* OR MA Nicaragua OR TI Nicaragua OR TI Nicaraguan* OR MA Panama OR TI Panama OR TI Panamanian* OR MA Paraguay OR TI Paraguay OR TI Paraguayan* OR MA “Peru” OR TI Peru OR TI Peruvian* OR MA “Uruguay” OR TI Uruguay OR TI Uruguayan* OR MA “Venezuela” OR TI Venezuela OR TI Venezuelan* OR MA “Central America” OR TI “Central America*” OR MA “Caribbean Region” OR TI Caribbean* OR MA “South America” OR TI “South America*” OR MA “Latin America” OR TI “Latin America*” OR AB “Spanish speak*” OR AB Argentina OR AB Argentine* OR AB Argentinian* OR AB Bolivia OR AB Bolivian* OR AB Brazil OR AB Brazilian* OR AB Chile OR AB Chilean* OR AB Colombia OR AB Colombian* OR AB “Costa Rica” OR AB “Costa Rican*” OR AB Cuba OR AB Cuban* OR AB “Hispaniola” OR AB Dominican* OR AB Ecuador OR AB Ecuadorian* OR AB Ecuadorean* OR AB “El Salvador” OR AB Salvadorean* OR AB Salvadoran* OR AB Salvadorian* OR AB Guatemala OR AB Guatemalan* OR AB Honduras OR AB Honduran* OR AB Mexico OR AB Mexican* OR AB Nicaragua OR AB Nicaraguan* OR AB Panama OR AB Panamanian* OR AB Paraguay OR AB Paraguayan* OR AB Peru OR AB Peruvian* OR AB Uruguay OR AB Uruguayan* OR AB Venezuela OR AB Venezuelan* OR AB “Central America*” OR AB Caribbean* OR AB “South America*” OR AB “Latin America*”) AND (MA “United States” OR TI “United State*” OR TI “USA” OR MA “North America” OR TI Appalachia* OR TI “great lakes” OR TI “mid atlantic state*” OR TI “mid atlantic region*” OR TI “middle atlantic state*” OR TI “middle atlantic region*” OR TI “midwestern us” OR TI “midwestern state*” OR TI “midwest state*” OR TI “midwest us” OR TI “great plains” OR TI “heartland” OR TI “new england” OR TI “northeastern us” OR TI “northeastern state*” OR TI “northeast state*” OR TI “northeast us” OR TI “pacific northwest” OR TI “northwest* us” OR TI “northwestern state*” OR TI “northwest state*” OR TI “pacific state*” OR TI “southeast state*” OR TI “southeastern state*” OR TI “southeast us” OR TI “southeastern us” OR TI “southern state*” OR TI “southern us” OR TI “southwest state*” OR TI “southwestern state*” OR TI “southwest us” OR TI “southwestern us” OR TI “deep south” OR TI “black belt” OR TI “rust belt” OR TI “District of Columbia” OR TI “Washington DC” OR TI Alabama OR TI Alaska OR TI Arizona OR TI Arkansas OR TI California OR TI Colorado OR TI Connecticut OR TI Delaware OR TI Florida OR TI Georgia OR TI Hawaii OR TI “Hawai i” OR TI Idaho OR TI Illinois OR TI Indiana OR TI Iowa OR TI Kansas OR TI Kentucky OR TI Louisiana OR TI Maine OR TI Maryland OR TI Massachusetts OR TI Michigan OR TI Minnesota OR TI Mississippi OR TI Missouri OR TI Montana OR TI Nebraska OR TI Nevada OR TI “New Hampshire” OR TI “New Jersey” OR TI “New Mexico” OR TI “New York” OR TI “North Carolina” OR TI “North Dakota” OR TI Ohio OR TI Oklahoma OR TI Oregon OR TI Pennsylvania OR TI “Rhode Island” OR TI “South Carolina” OR TI “South Dakota” OR TI Tennessee OR TI Texas OR TI Utah OR TI Vermont OR TI Virginia OR TI Washington OR TI “West Virginia” OR TI Wisconsin OR TI Wyoming OR TI “Western Hemisphere” OR MA “Americas” OR TI America* OR AB “United State*” OR AB “USA” OR AB Appalachia* OR AB “great lakes” OR AB “mid atlantic state*” OR AB “mid atlantic region*” OR AB “middle atlantic state*” OR AB “middle atlantic region*” OR AB “midwestern us” OR AB “midwestern state*” OR AB “midwest state*” OR AB “midwest us” OR AB “great plains” OR AB “heartland” OR AB “new england” OR AB “northeastern us” OR AB “northeastern state*” OR AB “northeast state*” OR AB “northeast us” OR AB “pacific northwest” OR AB “northwest* us” OR AB “northwestern state*” OR AB “northwest state*” OR AB “pacific state*” OR AB “southeast state*” OR AB “southeastern state*” OR AB “southeast us” OR AB “southeastern us” OR AB “southern state*” OR AB “southern us” OR AB “southwest state*” OR AB “southwestern state*” OR AB “southwest us” OR AB “southwestern us” OR AB “deep south” OR AB “black belt” OR AB “rust belt” OR AB “District of Columbia” OR AB “Washington DC” OR AB Alabama OR AB Alaska OR AB Arizona OR AB Arkansas OR AB California OR AB Colorado OR AB Connecticut OR AB Delaware OR AB Florida OR AB Georgia OR AB Hawaii OR AB “Hawai i” OR AB Idaho OR AB Illinois OR AB Indiana OR AB Iowa OR AB Kansas OR AB Kentucky OR AB Louisiana OR AB Maine OR AB Maryland OR AB Massachusetts OR AB Michigan OR AB Minnesota OR AB Mississippi OR AB Missouri OR AB Montana OR AB Nebraska OR AB Nevada OR AB “New Hampshire” OR AB “New Jersey” OR AB “New Mexico” OR AB “New York” OR AB “North Carolina” OR AB “North Dakota” OR AB Ohio OR AB Oklahoma OR AB Oregon OR AB Pennsylvania OR AB “Rhode Island” OR AB “South Carolina” OR AB “South Dakota” OR AB Tennessee OR AB Texas OR AB Utah OR AB Vermont OR AB Virginia OR AB Washington OR AB “West Virginia” OR AB Wisconsin OR AB Wyoming OR AB “Western Hemisphere” OR AB America*)) AND (TI “older adult*” OR TI frail OR TI aged OR TI elderly OR TI elder OR TI senior* OR TI gerontology OR TI geriatrics OR TI geria* OR MA Aged OR MA Frail Elderly OR MA Geriatrics OR AB “older adult*” OR AB frail OR AB aged OR AB elderly OR AB elder OR AB senior* OR AB gerontology OR AB geriatrics OR AB geria*)

### 2.3 Stage 3: study selection

All articles retrieved from database searches will be exported to the Covidence application (13). Following this, duplicate articles will be removed. Titles and abstracts of articles will be screened to determine relevancy based on the inclusion and exclusion criteria. A more detailed review of each article will be conducted for final inclusion. Furthermore, backward searches will be conducted by examining the reference lists of included articles, using the same selection process for articles identified during this stage. Two team members will conduct the literature search (JL and MJD), relevant articles will be identified and reviewed by both team members, and data will be extracted. Both team members (JL and MJD) resolved discrepancies through discussion to ensure consistency, and any unresolved disagreements were addressed by consulting with the third team member (CB). The criteria for including a full-text article were established beforehand with input from the stakeholder team. The criteria for participants, interventions, outcomes, context, and study design are summarized in Table 2.

- Population: the population of interest will consist of older Hispanic/Latino adults aged 60 and above living in the United States. The terms “Hispanic” and “Latino” are pan-ethnic terms often used interchangeably, referring to “individuals of Mexican, Puerto Rican, Salvadoran, Cuban, Dominican, Guatemalan, and other Central or South American or Spanish culture or origin” (14, 15). Throughout the scoping review, we will use the term Hispanic/Latino to refer to our population of interest.
  In many countries, older adults are typically considered to be 65 and older, though 60 may be more appropriate in certain contexts. Research evidence shows inconsistency in the ages of older participants (16). Therefore, in this scoping review, we focus on older adults aged 60 and older to gain a better understanding of older Hispanic/Latino adults and fall prevention, where research and literature are already limited.
- Interventions: studies describing fall prevention interventions (i.e., assessment, activities, programs, or strategies) targeting Hispanic/Latino older adults will be eligible for inclusion.
- Context and study design: while there will be no specific time limitation, studies published within the past 10 years will be prioritized to ensure relevance. Only studies published in the United States and written in English will be included in the review. Interventions delivered in healthcare or community-based centers will be considered, including primary care settings such as clinics, community hospitals, and nursing homes/assisted living, as well as community care settings, including senior and community centers, churches, and virtual groups. The inclusion of studies was not restricted by their design. All studies examining outcomes following the implementation of a fall prevention intervention using quantitative, qualitative, or mixed methods designs will be eligible for inclusion. Quantitative studies may include randomized and non-randomized controlled trials, and before-after designs (with or without control groups). Qualitative studies exploring participants’ views or experiences of an intervention, including different qualitative designs (e.g., grounded theory), will be considered. Pilot studies are included, while conceptual articles, protocol papers, reviews, case studies, and quality improvement studies are excluded. Quality assessment shall be done using the Mixed Method Appraisal Tool (MMAT) (17).
- Outcomes: this scoping review focuses on identifying the range of outcomes measured in the fall interventions rather than on specific results. We want to understand which outcomes researchers consider most important, not to evaluate how effective interventions are. By mapping these outcomes, we aim to give a clear overview of the current evidence and point out any gaps or patterns in outcome selection for future research. This approach will help expand our understanding of fall prevention for Hispanic/Latino older adults and maximize the effectiveness of evaluating future interventions.

**Table 2:** Study selection criteria.

Population	Intervention	Context	Outcome
Hispanic/Latino older adults aged 60 and above living in the United States.	Studies describing interventions (i.e., activities, programmed or strategies) targeting older Hispanic/Latino adults	Studies published in English (preferably within the past 10 years) with interventions delivered in healthcare or community settings	Focus on identifying a range of outcomes measured in the fall interventions, rather than focusing on specific results.

### 2.4 Stage 4: charting the data

The data will be extracted following the guidelines set by Arksey and O’Malley (8–10). Through collaboration with all team members, a data extraction form will be developed using Covidence. All potentially relevant sources will be retrieved and uploaded into Covidence. This specialized web-based instrument enhances researchers’ search efforts to gather all relevant details about a specific topic or research question, extract and categorize data, and produce various forms (18).

The extracted data will include the following information: study aims, methods, author(s), details of the intervention, usage of Hispanic/Latino terminology, definitions provided for Hispanic/Latino, identified gaps, year of publication, type of intervention (including any comparators), intervention model, duration of the intervention, sample characteristics, location, outcomes, and key findings. After both reviewers independently extract data, they will reach a consensus for each article. They will then export consensus data to an Excel spreadsheet and examine the extracted data for completeness, updating missing information as needed. Two team members, JL and MJD, will independently conduct data extraction. A third team member, CB, will then compare their results. Any inconsistencies in the data extraction process for each study will be resolved with the help of this third team member.

### 2.5 Stage 5: collating, summarizing, and reporting the results

Covidence and Excel spreadsheets will be used to develop a descriptive numerical summary of included studies and thematically analyze eligible articles through qualitative analytical techniques. Analysis will be performed to identify key concepts and themes across articles through an iterative process of combining, categorizing, and comparing information. Existing frameworks and models (19, 20) will be utilized to characterize the fall prevention interventions found in the literature. Tables, figures, and other data displays will be generated as needed to complement and substantiate the result of the study.

### 2.6 Stage 6: consultation

This scoping review protocol will engage relevant stakeholders. The stakeholders who helped develop and review the research questions and proposed search terms will be important in interpreting and sharing the project’s findings. The integration of stakeholders is further discussed in Discussion and Dissemination.

### 2.7 Ethics

This study involved no human participants and will utilize data from previously published articles. An exemption from review was received from the University of Central Florida (STUDY00007458). The review protocol has been registered through the Open Science Framework (https://doi.org/10.17605/OSF.IO/ZX3M8).

## 3 Discussion and dissemination

The objectives, rationale, methods, and design for this scoping review project that will examine the state of the science related to fall prevention among Hispanic/Latino older adults are described in this paper. This review seeks to examine the scope and nature of research studies on fall interventions targeting Hispanic and Latino older adults. To achieve this aim, a dynamic team of researchers and community partners was established. Stakeholders include Hispanic/Latino older adults, community leaders, and an aging fall prevention expert who will be collaborating to ensure relevance, quality assurance, and dissemination of the results.

Similar to other review papers, this project may exclude equally relevant papers written in foreign languages or articles that may be found outside the wall of indexed article databases identified in the methods section. Our members will perform spot-checking and hand-searching techniques to address this limitation. Also, inter-reviewer reliability shall be reported to avoid potential bias in the screening and assessing published articles.

As far as we are aware, this work will be the initial effort to comprehensively describe the scope and characteristics of fall interventions targeting Hispanic/Latino older adults. This includes the models utilized, cultural tailoring, and the outcomes these interventions aim to modify.

Our stakeholders will play a crucial role in interpreting the review findings and sharing them within Hispanic/Latino communities to ignite discussions about fall prevention. In our stakeholder meetings, we will delve into these findings while exploring future research and clinical opportunities. Engaging with stakeholders not only enhances the cultural relevance of our review process but also ensure review finding are culturally relevant and reflect older Hispanic/Latino community experiences. We will document key themes and actionable insights from these discussions in field notes and summary reports, which will provide a solid foundation for our future efforts. The insights gathered from community stakeholders will significantly enhance the development of fall prevention interventions, allowing us to align our initiatives more closely with the values and needs of the community. This approach will help us create fall prevention programs that are tailored specifically to the culture of Hispanic/Latino older adults in our area.

Our review will be published in peer-reviewed journal and presented at conferences, including the Gerontological Society of America, to contribute to the field of community-engaged research. Additionally, we plan to explore how the results of stakeholder engagement meeting can offer valuable insights for communities focused on fall prevention in a future manuscript, highlighting practical applications and community-driven strategies. We aim to submit the review to an open-access journal, making it accessible to scholars, Hispanic/Latino older adults, their families, and the general public.

Ultimately, stakeholder involvement enhances community buy-in, effectiveness, and sustainability of fall prevention efforts. We hope their input will help identify key priorities for developing and delivering culturally tailored fall prevention strategies for Hispanic/Latino older adults in the United States, fostering trust and equitable health outcomes.
